# Tuberculosis in Children with Rheumatic Diseases Treated with Biologic Disease-Modifying Anti-Rheumatic Drugs

**DOI:** 10.31138/mjr.32.4.290

**Published:** 2021-12-27

**Authors:** Chengappa Kavadichanda, MB Adarsh, Sajal Ajmani, Ilaria Maccora, S Balan, AV Ramanan, Vikas Agarwal, Latika Gupta

**Affiliations:** 1 Department of Clinical Immunology, Jawaharlal Institute of Postgraduate Medical Education and Research, Puducherry, India,; 2 Department of Medicine, Government Medical College, Kasaragod, India,; 3 Arthritis and Rheumatology Clinic, Delhi,; 4 University of Florence, School of Health Science, Rheumatology Unit, Meyer Children’s University Hospital, Florence, Italy,; 5 Department of Paediatric Rheumatology, Amrita Institute of Medical Sciences, Kochi, India,; 6 Bristol Royal Hospital for Children & University of Bristol, United Kingdom,; 7 Department of Clinical Immunology, Sanjay Gandhi Postgraduate Institute of Medical Sciences, Lucknow, India

**Keywords:** tuberculosis, biologics, rheumatology, paediatrics

## Abstract

Chronic rheumatic diseases entail the use of biologics in children. Immunosuppressive effects of drug therapy put children at risk of various infections including tuberculosis (TB). Even though TB is a major concern among individuals on biological DMARDs, the incidence and distribution among children on these drugs is not known. Hence, we performed a literature search to ascertain the prevalence of tuberculosis amongst children with rheumatic disorders treated with biological agents. Articles available on MEDLINE and SCOPUS published on or after January 1, 2010 to 1 October 2019 were reviewed and collated. We found that published data on TB infections in children with rheumatic disorders on biologics is scant even from regions with highest TB burden. Tuberculosis was reported on occasion (0–5 cases per country) in the developed world with most reports being from Turkey. While most of the retrospective studies suggest that TB risk is minimal in the paediatric rheumatology patients, prospective studies suffer from a short observation period. Most registries focus on response to therapy rather than complications. In this review we have then discussed about the variation in screening strategies for latent TB and the role of bacille Calmette-Guerin (BCG) vaccination. Based on the dearth of data and inconsistency in data collection, we propose a way forward in the form of establishing well-designed long-term prospective national registries from countries with high background prevalence of TB with focus not only on treatment efficacy but also on adverse events and infections.

## INTRODUCTION

Despite the advent of glucocorticoids and immunosuppressive therapies, chronic rheumatic diseases of childhood such as Juvenile Idiopathic Arthritis (JIA), Systemic Lupus Erythematosus (SLE), Idiopathic Inflammatory Myositis (IIM), Auto-inflammatory Syndromes (AIS)and Paediatric Vasculitis (PV) result in significant morbidity, and, at times, even mortality.^1–3^ In the developing world, infections are the leading contributors to such morbidity. Tuberculosis (TB) is one such infection, which remains a particular challenge in these parts of the world.^4^ The emergence of drug-resistant tubercular strains and polypharmacy, in the setting of chronic illnesses further compounds the problem.^5^

Recent estimates suggest the prevalence of TB in India to be 3.2 cases per thousand population.^4^ The presence of rheumatic disorders (RDs) entails treatment with glucocorticoids and immunosuppressive drugs for prolonged periods, more so in cases of lupus, vasculitis and myositis. Some patients with JIA, lupus, vasculitis, and, rarely, IIM, also have underlying antibody deficiencies or complement pathway defects, further increasing their infection risk. Over the past years, there have been efforts towards decreasing usage of glucocorticoids in rheumatic disorders and advocating rational use of immunosuppressive agents. In addition to this, public health initiatives have attempted to address the issues of adequate treatment of TB.^2^ The changing dynamics of therapeutic practices could have a bearing on the prevalence of TB in these diseases, and also influence the ways this problem can be addressed. Thus, it is important to understand the prevalence, risk factors, and outcomes of TB infection among children with RDs on biologics. In this review, we have performed a literature search on the prevalence, screening strategies, and global reporting patterns of TB across various studies among children with RDs on biological DMARDs. We have then summarised the available literature and discussed the possibilities that could explain our findings. Finally, we have suggested the way ahead to obtain more robust information from underrepresented countries.

## REVIEW STRATEGY

The search strategy for writing review articles as proposed by Gasparyan et al. was followed.6 Articles available on MEDLINE and Scopus, published on or after January 1, 2010, until October 1, 2010 were reviewed using search words “juvenile” and “dermatomyositis” and “biologics” (n=71); “paediatric” AND “Lupus” AND “biologics” (n=81); “paediatrics” AND “Vasculitis” AND “Biologics” (n=55).

In addition, for the literature review on registry data in paediatric rheumatology, Scopus searches were conducted combining “registry” with each of the following: “paediatric” AND “Lupus” (n=100), “juvenile” and “myositis” (n=40); “juvenile” and “arthritis” (n=368); biologics” AND “Rheumatology” (n=359) and “Autoinflammatory” AND “syndromes” (n=50).

**Figure 1.**
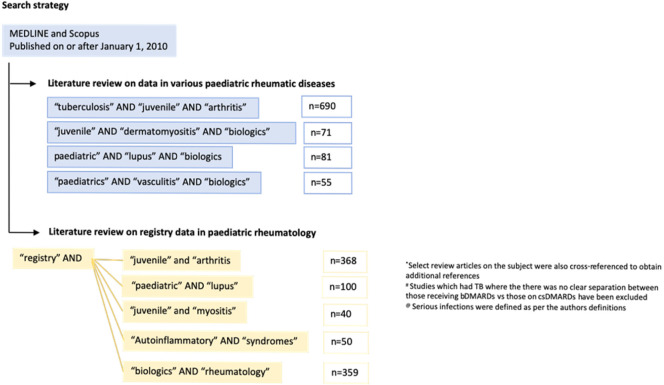
Number of articles obtained after searching through MEDLINE and Scopus.

Also, select review articles on the subject were cross-referenced to obtain additional references. **Figure 1** summarises the search results.

Articles with data on outcomes in children of treatment with biologics were included. Review articles, systematic reviews, case reports, and articles without data in children, and those in languages other than English, and where full-text was not available were excluded. Congress abstracts did not feature in the searches. Studies which had TB where there was no clear separation between those receiving bDMARDS vs those on csDMARDs were excluded. Serious infections were defined as per the publishing author’s definitions. The Zotero software, an open-source tool, was used for references management and citations.

## SELECTION OF ARTICLES

### Screening by title

The Scopus searches were imported into Zotero, and articles were first screened by title by one author, and those without relevance, systematic reviews, meta-analysis, narratives, and in languages other than English were removed (**Figure 1**). The exact process of data extraction is elaborated in the supplementary material.

### Juvenile Idiopathic Arthritis and Tuberculosis

Juvenile idiopathic arthritis is a chronic rheumatic disorder consisting of polyarticular (rheumatoid factor positive and negative), oligoarticular, systemic-onset JIA, enthesitis-related-arthritis, psoriatic and undifferentiated subtypes. The occurrence of infections is known and associated with poor outcomes.^7^ Tuberculosis is a chronic infection that can result in significant morbidity and mortality in children with JIA.^8^

**Figure 2.**
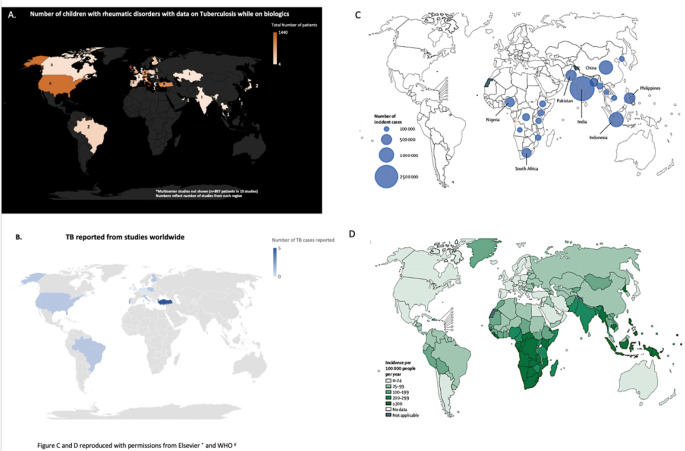
Global distribution of cases. **A.** Data available on children with paediatric rheumatic disorders on biologics. **B.** Number of tuberculosis cases reported from studies summarised in **Figure 2A**. **C.** Number of incident tuberculosis cases worldwide^*^. **D.** Global incidence of tuberculosis per 10000 people^#^.

Data in JIA consists of mixed cohorts of various subtypes of arthritis. Interestingly, most series report no occurrence of Tuberculosis (**Tables 1, 2 and 3**). Tuberculosis has been reported in four prospective studies, involving 2 each from Turkey and Portugal, and 1 each from Brazil and a multicentre trial. The follow-up duration in these studies ranged over 1–5 years. Of the various biologic registries screened, the only two cases of Tuberculosis reported are from Turkey. This is in contrast to minimal or no reports of Tuberculosis from UK, most European countries (France, Germany, Italy, and Greece) and Canada. The general prevalence of tuberculosis in Turkey is26/100,000 (2005). Brazil has one of the highest TB burdens with over 70,000 incident cases per year (**Figure 2D**). Portugal has the highest TB prevalence in Western Europe at 23 per 10,000 population, which resonates with the 2 cases reported of two studies in 232 patients.^9^

**Table 1.** Data of tuberculosis in retrospective studies of patients with Juvenile arthritis on biologics.

Retrospective					
**Country**	Italy	Taiwan	Italy	Turkey	Canada
**Year**	2012	2015	2017	2010	2015
**Author**	Bracaglia^42^	Hsin^29^	Favalli^43^	Ayaz^44^	Hugle^45^with a median follow-up period of 7.2 years. Prospective data was collected according to a standardized protocol. Outcomes examined were TEC, TAJC, markers of inflammation (ESR, CRP
**Tuberculosis-No of patients**	0	1	0	0	0
**Type of article/paper**	Retrospective analysis of a cohort	Nested case control analysis of Taiwan National Health Insurance Research Database	Data extracted from local registry looking at the causes for anti TNF withdrawal	Retrospective chart review	Retrospective chart review
**Type of JIA**	N=25	N=111	N=360	N=36	N=16
**PA-RF+**	3(12 )	NA	31	6 (16.7)	0
**PA-RF -**	1(4)	NA	75	0	0
**OA**	3 (12)	NA	101	0	0
**OA Extended**	9(36)	NA	70	3 (8.3)	0
**ERA**	0	NA	26	12 (33.3)	16
**SJIA**	8(32)	NA	48	14 (38.9)	0
**PsA**	1(4)	NA	9	1 (2.8)	0
**Undiff**	0	NA	0	0	0
**Duration of follow-up (Median)**	10 months (2–41)	3.49 ± 1.79 years (Mean)	10 years	36 months (range 4–216 months)	7.2 years (4.5 – 12.1) 117.1 patient-years
**N total whose complete data is available**	25	111	354	36	16
**Drug**	ETN 25	Anti TNF (Mainly ETN)-111	IFX-89 ETN- 205 ADA-66	ETN-36	IFX, ADA and ETN combinations-16 IFX alone 8 ETN alone 5 ETN followed by ADA 1 IFX followed by ADA 1 IFX followed by ETN, then by ADA 1
**Biologic Doses received**	ETN0.8–1 mg/kg once weekly	Anti TNF (No data on individual drugs)	NA	NA	NA
**Duration of biologic treatment**	23 months (mean)	Max 8 years	NA	11.5 months (3–48 months)	NA
**Concomitant drugs**	MTX 24 (96%) CYS 3 (1.6%)	MTX (number NA)	NA	NA	NA
**Steroids**	10 952.6%)	NA	NA	NA	NA
Brazil	Turkey	Poland	Italy	India	Turkey
2017	2011	2011	2016	2016	2016
Brunelli^46^	Kilic^47^	Żuber^48^	Verazza^49^	Saini^50^	B A Atikan^51^
0	0	1	1	0	0
Retrospective cohort that included JIA patients eligible to anti-TNF therapy	Retrospective chart review	Polish registry data collected between January 2003 and March 2010	Retrospective Multicentre Italian Paediatric Rheumatology Study Group led chart-based review	Research letter	Retrospective chart review of patients who were given biologicals and had received BCG vaccines
N =69	N=132	N=188	N=1038	N=10	N=71
9 (13)	73, (50.7)	13 (7)	50 (4.8)	3	18
22 (32)		79 (42)	329 (31.7)		
0	22, (15.3)	27 (14)	139 (13.4)	0	5
12 (17)		30 (16)	325 (31.3)	0	
6 (9)	14, (9.7)	1 (0.5)	48 (4.6)	0	20
19 (28)	19, (13.2)	28 (15)	106 (10.2)	7	23
1 (1)	4, (2.8)	2(1)	34 (3.3)	0	5
0	0	8 (4)	7 (0.7)	0	0
2.9 years(0.3–24.6)	5.86 ± 3.77 years	Mean ±SD 52 (41.7) months Range- 2–183 months	2.1 (0.6–5.5)years	11 (range 4–41) months	3 years
69	132	39	NA	10	NA
ADA-12 ETN-35 ETN switched to→ ADA 17 ADA → ETN 2 ETN → IFX 1 IFX → ETN → ADA 1 ETN → ADA → IFX 1	ETN 115 IFX 17 ETN + IFX 6 IFX + ADA 4 ETN + ADA 2,	ETN-188	ETN-1038	ETN-5 TCZ-5 ABA-1 (switched from TCZ)	ETN-41 ADA-21 CANA-5 TCZ-4
NA	NA	NA	NA	NA	NA
ADA-21.4 (2.3–73.5) ETN-25.6 (0.5–95) IFX-1.9 (0.03–8.5)	NA	393 patient-years	2.1 (0.6–5.5)	11 (range 4–41) months	3 years
MTX-60 (87) Dose 25 (5–50) LEF- 23 (33) CYS-13(19)	NA	37(95)	Mtx749 (72.2)	NA	NA
31 (45)	NA	35(92)	267 (25.7)	NA	NA

ADA: Adalimumab; ETN: Etanercept; IFX: Inflixim; ABA: Abatacept; CER: Certolizumab; GOL: Golimumab; JIA: Juvenile idiopathic arthritis; AZA: Azathioprine; MTX: Methotrexate; CYS: Cyclosporine; LEF: Leflunomide; SSZ: Sulfasalazine; CAN: Canakinumab; RTX: Rituximab; ANK: Anakinra; TCZ: Tocilizumab; JIA: Juvenile idiopathic arthritis; PA: Polyarticular; SJIA: Systemic onset juvenile idiopathic arthritis; ERA: Enthesitis related arthritis; OA: oligoarticular; Undiff: Undifferentiated.

**Table 2.** Data of tuberculosis in prospective studies of patients with juvenile arthritis on biologics.

Prospective								
**Country**	Turkey	Portugal	Germany	USA and Canada	The Netherlands	Multicentre-Europe, Latin America and USA	Multicenter- 19 countries	Japan
**Year**	2018	2016	2015	2009	2009	2010	2015	2011
**Author**	Aygun^52^	Mourão^53^	Horneff^54^	Giannini^55^	Prince^56^	Ruperto^57^	Constantin^58^	Imagawa^59^
**Tuberculosis-No of patients**	2 (1-ETN) (1-ADA)	2 1- ADA 1-ETN (Skin test conversion)	0	0	0 (But 1 had TB after switching to IFX- Reported as a case report)	0	0	0
**Type of article/paper**	Single centre cohort	From Reuma.pt. database	Phase III Randomise Double-Blind Study	Phase IV, open-label, multicenter registry	Multi-centre (Dutch national registry)	Long-term, open-label extension phase of a double-blind, randomised, controlled withdrawal trial	Phase IIIb, open label, multicentre study	Open-labelled multicentre study
**Type of JIA**	N =307	N=227	N=41	N=397	N=146	N=186	N=127	N=19
**PA-RF+**	18 (5.9)	36 (17.5)	0	351	11 (8)	38 (20%)	0	9
**PA-RF -**	85 (27.7)	48 (23.3)	0	0	55 (38)	84 (44)	0	8
**OA**	100 (32.6)	20 (9.7)	0	0	0	0	0	0
**OA Extended**		33 (16)	0	Included as PA	28 (19)	27 (14%)	60(47.2)	2
**ERA/SpA**	42 (13.6)	31 (15.1)	20	0	5 (3)	0	38(29.9)	0
**SJIA**	52 (16.9)	28 (13.6)	0	45	39 (27)	37 (20%)	0	0
**PsA**	10 (3.3)	10 (4.8)	0	0	8 (5)	0	29(174)	0
**Undiff**	0	21 (9.8)	0	1	0	0	0	0
**Duration of JIA before biologics (Median, IQR)**	NA	13.7 (10.1) years	2.4 ± (2.1) years	58.1±44.5 ETN 40.7±41.7 ETN+MTX	4.1 years	1,069 days (range 168–1,457 days)	NA	4.7 yrs (1–17)
**Duration of follow-up (Median)**	12 months	At least 12 months	48 weeks	36 months (41% completed 36 months)	2.5 years per patient, (range 0.3 to 7.3 years)	589 days	96-weeks	48 weeks^*^ all except 2

Brazil	Finland	USA and EU	Japan	Multicentre	Multicentre	Germany	Multicentre – North America, South America and Europe
2017	2015	2014	2012	2008	2008	2015	
Brunelli^60^	Maarit Tarkiainen^61^	Kingsbury^62^	Imagawa^63^	Lovell^64^	Lovell^65^	Gerd Horneff^54^	Ruperto N^66^
1- With TCZ	0 1 case of MAC with ADA	0	0	0	0	0	1 Lung infiltrate with negative sputum AFB
Longitudinal data of patients with JIA receiving at least 8 weeks of biological agent documented between August 2004 to March 2016	Observational multicentre study	multicentre, open-label, phase 3b	single-arm, open-label, multicentre study	Multicentre open label extension trial^*^ Not classified as per modified ILAR	Randomised, double-blind, stratified, placebo-controlled, multicentre, medication-withdrawal study with a 16-week open-label lead-in phase, a 32-week double-blind withdrawal phase, and an open-label extension phase.	Phase III Randomised, Double-Blind Study	Randomised, Placebo-Controlled, Double blind Trial
N=107	N=348	N =32	N =25	N=58	n=171	N=20	N=117
52(48.6)	16 (4.6%)	1(3.1)	17(68)	5	40(23.4)	0	117
	175 (50.3%)	20(62.5)	8(32)	0	131(76.6)	0	0
19(17.8)	30 (8.6%)	0	0	34	0	0	0
	65 (18.7%)	8(25)	0	0	0	0	0
7 (6.5)	22 (6.3%)	0	0	0	0	20	0
28 (26.2)	19 (5.5%)	2(6.3)	0	19	0	0	0
1(0.9)	0	0	0	0	0	0	0
0	1(0.3%)	1(3.1)	0	0	0	0	0
Median 4.8 years (0.1–21)	NA	12.3 (9.3) mean months	4.7 (3.72) mean years	8 years	4.0±3.7 3.6±4.0 Years in ADA+MTX and ADA arms	2.4 ± (2.1) years	NA
median 3.0 years (0.15–11.5)	50.5 months (range 1.0154.7)	Max-120 weeks	60 weeks	58	48 weeks	48 weeks	52 weeks

Prospective								
**Exposure in patient years**	NA	706.92 patient- years	35.6 patient year	NA	436.1 patient years	NA	NA	NA
**N total whose complete data is available**	307	227	38	397	146	153	109	17
**Drug**	ETN 189 ADA 60 ANK 22 CAN 12 IFX 11 TCZ 13	ETN 157 ADA 29 ABA 8 TCZ 2 ANK 11 IFX 19 RTX 1	ETN 41	ETN-397 ETX only 103 ETN+ MTX -294	ETN-146	ABA-186	ETN-127	TCZ-19
**Biologic Doses received**	NA	NA	ETN treatment (0.8 mg/kg BW, maximum dose 50 mg/week)	ETN 0.8 mg/kg/week, maximum dose 50 mg	0.8 mg/kg once weekly	10mg/kg ABA q4W	ETN 0.8 mg/kg once weekly (QW; max dose, 50 mg) for up to 96 weeks	8 mg/kg TCZ every 4 weeks.
**Duration of biologic treatment**	42.11 ± 35.78 months (range 2–380 months)	4.5 (3.1) Years	48 weeks	NA	1.7 years (range 0.1 to 6.8 years)	NA	96 weeks	48 weeks
**Concomitant drugs**	NA	MTX- 170 patients SSZ 16	SSZ was allowed	MTX 294 Dose-12.6 ±5.3 (ETN) 16.9± 5.9(MTX+ETN)	MTX-113 (77)	MTX in 74% 57 (30%) Prior biologics	NA	MTX 100%
**Steroids (%)**	NA	NA	No	NA	90 (62)		NA	
**Other**	NA	NA				NA	NA	NA

398.3 patient-years (py): 179.1py for ETA, 92.5py for ADA, 21.7py for IFX, 77.8py for ABA and 27.2py for TCZ	710 py-ETN, 591PY-IFX, 188 PY-ADA, 8 PY-RTX, 5.3 PY-ANK, 6.4 PY-ABA, 6.4 PY- TCZ and 1.0 PY- GLM	45.1 PY	NA	NA	NA	29.9 patient year	NA
107	348	26	25	9	128	19	117
ETN ADA ABA TCZ IFX	ETN 213 IFX214 ADA94 RTX9 ANK-8 ABA-6 TCZ 4 GLM3	ADA31	ADA25	ETN58	ADA171	ETN 20	IFX-117
NA	NA	24 mg/m^2^ (maximum = 20 mg/dose) every other week up to 120 weeks	20 mg for patients weighing <30 kg, and 40 mg for patients weighing ≥30 kg) eow	ETN 0.8 mg/kg/week	fixed dose based on body weight (20 mg for patients weighing <30 kg, and 40 mg for patients weighing ≥30 kg) eow	NA	6mg/kg and 3mg/kg standard protocol
Median 3.0 years (0.15–11.5	NA	515 (245 ) days	24 weeks	318 PY	48 weeks	48 weeks	52 weeks and 38 weeks
NA	NA	MTX 27 (84.4)	MTX-20(80)	(100)	MTX-85(49.7)	NA	Mtxupto 15mg/m^2^
NA	NA	20(62.5)	NA	22 (38%)	NA	NA	NA
	NA	NA	NA	NA	NA	NA	NA

**Table 3.** Data from studies on cohorts/registries of children with Juvenile arthritis on biologics.

**Country**	Germany	UK	Multicenter member centres 32 countries	Germany
**Year**	2019	2011	2018	2014
**Author**	A Klein^67^	Southwood^68^	J Swart^32^	Schmeling^69^
**Registry Name**	BIKER	Biologics and New Drugs Registry (BNDR)	PharmaChild	German Biologics JIA Registry
**Tuberculosis-No of patients**	0	0	24 over all 14 on biologics Total 17 Tb in 14 patients on biologicals	0
**Type of article/paper**	long-term data from the German BIKER registry	Prospectively collected Data	Combined data form PharmaChild registry along with German and Swedish registries	The registry is a longitudinal multicentre observational study that has been maintained since 2000
**Biological agent**	ADA 584	ETN-483	ETN-3600 ADA- 1778 IFX- 705 CER- 33 GOL- 161 TCZ-633 ABA- 420 RTX 103 ANK- 339 CAN- 145	ADA-289
**Rheumatological condition**	JIA N=584	JIA N=483	JIA N= 8274	JIA N=289
**PA-RF+**	34 (5.8)	48 (9)	322 (3.9)	17 (6.2)
**PA-RF -**	203 (34.7)	157 (33)	2183 (26.4)	101 (34.9)
**OA**	42	11 (2)	2011 (24.3)	28 (9.6)
**OA Extended**	0	79 (16)	1060 (12.8)	68 (23.5)
**ERA**	98	38 (8)	924 (11.2)	39 (13.5)
**SJIA**	0	77 (16)	911 (11)	8 (2.7)
**PsA**	49	30 (6)	285 (3.4)	14 (4.8)
**Undiff**	11	36 (7)	578 (7.0)	14 (4.8)
**Unclassified**	0	7 (1)	0	0
**Duration of follow-up (Median)**	NA	NA	NA	NA
**Follow-up in patient years**	1082 patient-years (PY)	941 patient-years of follow-up		435.7 patient-years
**N total whose complete data is available**	584	483	5173	289

Germany	Hungary	The Netherlands	UK
2017	2011	2011	2019
Horneff^70^	Sevcic^71^	Otten^72^	Fleet^73^
BIKER Germany	National Institute of Rheumatology and Physiotherapy Registry: Hungary	Dutch Arthritis and Biologicals in Children Register	Biologics for Children with Rheumatic Diseases (BCRD) study
0	0	0	0
Cohort of Systemic JIA	Cohort	Cohort of children receiving Tumour Necrosis Factor-blocking Agents for in JIA-ERA—collected between 1999–2010	Cohort
ETN-143 TCZ-71 IL-1 Inhib-60	ETN- 72	ETN 20 ADA-2(1 switched) IFX-2 (1 switched)	RTX- 41
JIA-N=245	JIA-N=72	JIA-N=22	JIA-N=41
0	6(8)	0	13 (33)
0	36(50)	0	14 (35)
0	0	0	2 (5)
0	20(28)	0	9 (23)
0	3(4)	22	0
245	6(8)	0	1 (3)
0	1(2)	0	1 (3)
0	0	0	0
0	0	0	0
NA	12 months	14–28 months	177 days (IQR 109–398)
ETN-n = 143; 355.8PY TCZ-n = 72; 111.6PY n = 60; 116.8PY	NA	38.7 patient years	51 person-years
245		22	38

**Treatment duration and drugs**	15.1 ± 12.8 months ADA 15.2 ± 13.3 months ADA+ MTX	NA	ETN- 719 (300–1338) days ADA- 442 (174–927) days IFX- 425 (160–951) days TCZ-351 (126–742) days ABA- 342 (156–715) days ANK- 299 (94–837) days GOL- 270 (106–623) days CAN-351 (133–1032) days RTX-42 (24–87) days CER-166 (106–309) days	1.2 years (IQR 0.58–1.88) in Biologics naïve groups and 1.13 years (IQR 0.61–1.94) in previous biologic usage group
**Concomitant drugs**	MTX in 356 patients	NA	NA	MTX- 171 (59.2) LEF-13 (4.5) SSZ- 6 (2.1)
**Steroids**	NA	NA	NA	102 (35.3)

ETN-n = 143; 355.8PY TCZ-n = 72; 111.6PY n = 60; 116.8PY	ETN 0.4 mg/kg body weight twice weekly or ADA 40 mg every 2 weeks 12 months	NA	51 person-years
MTX-185	NA	MTX-17(77) SSZ-2 (9)	NA
176	NA	3 (14)	NA

Interestingly, a study from India which has one of the highest background prevalence of Tuberculosis in the world, reported no Tuberculosis though the follow-up duration was 11 months. Plotting data available from various studies in paediatric rheumatology on a world map reveals the distribution is primarily limited to regions with low TB prevalence (**Figure 2A**). There is sizeable risk of confirmation biases regarding the safety of biologics resulting from absence of data from high TB incident parts of the world (**Figure 2 C**).

On the contrary, in adults, there are reports of greater tuberculosis on anti-TNFs, with the risk being highest with IFX(cumulative incidence 0.5% within the first 500 days of registration) as compared with ETA (0.2%).^10,11^ It is worthwhile considering if BCG vaccination practices in children could explain differences between children and adults. Usage of biologics also induces an immunosuppressant state, and there is known risk of higher extra-pulmonary forms of TB in such a setting.^12^ Diagnosing these could be a challenge, particularly so in the absence of a robust biomarker for extrapulmonary forms of tuberculosis.^13^

### Juvenile Lupus and Tuberculosis

Data on tuberculosis in paediatric lupus is scant, being limited to 7 retrospective and 2 prospective studies (**Supplementary Table S1**). While most described the use of Rituximab, one prospective study on Belimumab featured 39 cases over 6 months of follow-up. The maximum duration of follow-up was 3 years and the largest series of 104 was from the United States in 2015. Whilst none of the series reported any tuberculosis, the largest series had overall 22 infections, of which 20 were major infections. Of note, most patients were on concomitant immunosuppressants or steroids during the study period. However, literature is replete with case reports of tuberculosis in lupus.^14^ We have previously found TB in 6% of children with LN.^5^ Thus, poor tuberculosis reporting could be from use of biologics in patients with less severe disease (minor organ manifestations), early mortality or underreporting. Previously use of high-dose cyclophosphamide (CYC) has been identified as a risk factor for infections in lupus.(15) The risk of infections could possibly be lower with biologics such as belimumab and RTX but this needs to be confirmed in larger studies.(16)

### Juvenile inflammatory myositis and tuberculosis

Out of the various studies on inflammatory myositis, none looked at data on Tuberculosis specifically (**Supplementary Table S2**) suggesting dire need to collect information relevant to this in future studies. On the other hand, we have 4 papers previously describing high prevalence of tuberculosis in myositis, suggesting the need for careful assessment of this aspect in prospective cohorts with longer term follow-up.^17–20^ We have described TB in 17.1% children from India with myositis (n=35, unpublished data). Unfortunately, biologics use is limited in this part of the world due to insurance policies and consequent financial constraints further leading to dearth of data.

### Juvenile Vasculitis and Tuberculosis

Data on paediatric vasculitis is scant, being limited to 5 retrospective series, most being on Behcet’s disease, Takayasu’s arteritis, and Polyarteritis nodosa from Turkey, UK and Canada, overall reporting 35 cases (**Supplementary Table S3**). No serious infections were reported over the longest study period of 2.1 years.

### Autoinflammatory syndromes and Tuberculosis

Although there is emerging data from registries including the Eurofever registry on various auto-inflammatory syndromes, most focus on treatment regimens and response to therapy with dearth of data on infections. In the limited studies available (**Supplementary Table 4**), no Tuberculosis was reported.^21–23^

**Table 4.** Summary of available data that could be analysed for tuberculosis incidence in paediatric rheumatology with various biologics.

**Drug/disease**	JIA	Lupus	Myositis	Autoinflammatory syndromes	Vasculitis
**Infliximab**	783(A) 547(B)	0	0		10(C)9(B)
**Adalimumab**	2925 (A) 489(B)	0	0		1(B)11(C)
**Etanercept**	6974 (A) 2019(B)	0	0		1(C)
**Certolizumab**	70 (A)0(B)	0	0		0
**Golimumab**	385 (A) 3(B)	0	0		0
**Rituximab**	210 (A) 51(B)	75(B)	48(E) 185(C)		3(C)
**Belimumab**	0(A) 0(B)	39(B)	0		0
**Anakinra**	810 (A) 63(B)	0	0	29(A) 27(B)1(D)	0
**Canakinumab**	241 (A)	0	0	4(A)109(E)	0
**Tocilizumab**	998 (A)	0	0		2(B) 9(C)
**Abatacept**	521 (A)	0	0		0
**Combination of anti TNFs**	3(A)	0	0		0

A: Registry data; B: Cohort; C: Case series; D: Anecdotal reports; E. Trials.

### Choice of biologics and risk of TB in children

Children with rheumatic disorders might be predisposed to Tuberculosis due to the intrinsic mechanism of action of biologics, anti-TNFs in particular, as they target TNF-α, the key cytokine for the Th1 axis. Experience from the biologic usage in adult rheumatic diseases has shown higher chances of TB reactivation with anti TNF agents. We identified 37 episodes of TB in 34 patients out of the 14,218 patients treated with anti TNF agents. In the non-TNF biologic group, a single case of TB has been reported with tocilizumab (OR-6.92 95% CI 0.95,50.56) (**Table 4**). Anti-TNF therapy may not be a cause for TB reactivation among children with autoimmune diseases on biological agents. The role of TNF in controlling TB infection is reflected by the mice models deficient in TNF. These rodents are unable to control M. tuberculosis infection and form granulomas in their lungs.^24^ TNF-α is required in the protective immune response against M.tuberculosis (MTB) in mice.^25^ TNF is an important signal for macrophage activation, in conjunction with IFN-γ. This cytokine has a key role in the immune responses to MTB, because it is involved in multiple processes, such as macrophage activation and cell recruitment to the sites of infection (natural killer cells, granulocytes, fibroblasts, and T cells), which either leads to granuloma formation or kills the pathogen. Furthermore, it activates CD8+ T cell that could directly kill the bacteria, TNF-α additionally activates CD8+ cytotoxic T cells (CTLs) that may be important because these cells release granulysin and directly kill intracellular bacteria. TNF-α also promotes the maturation of monocytes to dendritic cells (DCs) and/or macrophages, inducing the antigen presentation of intracellular mycobacteria. TNF-α produced in a local infection site allows macrophages, natural killer (NK) cells and γδ T cells gather at the infection site and bring their activation.^26^ The activated CTL cells have the ability to produce perforin protein and TNF-α by itself, which guide TB-infected monocytes to apoptosis, which involves intracellular living TB bacilli, and to induce the autophagy of infected cells via activated.^24^

The other possibility is an increased risk due to the presence of an autoimmune disease. The risk of infections seems to be increased in rheumatic diseases not only from the drugs used, but also the presence of T lymphocytes dysfunction and cytokine imbalance. Azfar et al. have shown that lupus patients have suppressed reactive oxygen species and tumour necrosis factor-alpha activity in human monocytes in response to mycobacterium TB.^27^ Previously, the risk of TB has been shown to be increased in children with JIA independent of the use of anti-TNFs.^28,29^ However, in this study, the risk of TB was equal to the general population for children who either received anti-TNFs, or non TNF biological agents. This in sharp contrast to numerous other reports of TB in adults, suggesting that anti-TNFs might be safer in children than reported adults. Though this could also be attributed to smaller numbers in subgroup analysis, and remains to be confirmed.

Presence of other infections can be risk factors for subsequent infections, though there is limited data from the current searches to substantiate that. One of the children who had CMV infection also had TB. In addition, primary immunodeficiencies such as X-linked agammaglobulinemia can mimic JIA and put the children at risk for infections.^30,31^

### Causes for low TB in children in current data set

#### Low numbers due to studies in regions with low incidence of TB

The number of studies from the various countries along with the reported number of TB cases, are plotted on a world map (**Figure 2A,B**). This pictorial view of the geographic distribution of the data obtained shows the stark distinction where most of the studies are concentrated in the affluent European and North American countries. Understandably, the reports of TB (**Figure 2B**) available are also from these countries. It is evident that the countries with the highest burden of TB (**Figure 2C**) have hardly any data on the biological use in children with RDs. Our literature search has brought out the inequalities in data availability across the world, and this has resulted in the probable assumption of low risk of TB among children with RDs on bDMARDs. Although the data review here suggested limited cases of TB on biologics, closer examination of the worldwide prevalence of TB makes paucity of data to be a possibility. The data from the PharmaChild registry had 17 episodes of TB in 14 children receiving biologicals for JIA.^32^ All the cases were reported from children on TNF inhibitors. TB was most reported from Asian patients - 52%, followed by 37% among the European patients, and 11% in the children treated at the centres in the USA. Since the registry covered 32 countries across the globe, the data seem to point at the fact that the low incidence of TB in other studies seen in **Figure 2**, is due to a concentration of studies from countries which are not endemic to TB.^33^ Studies from areas with moderate TB burden like Turkey and Brazil did report tuberculosis (**Figure 2**).

#### Low number of TB cases as consequence of the methodologies used to collect data

Moreover, the low reporting of adverse events could be relevant to the kind of data collected. Many articles in paediatric rheumatology focus on response to therapy. Thus, data recording of infections takes a backseat. Two cases of tuberculosis were reported in a single study from Turkey, with the use of etanercept and adalimumab, which focused on collecting infection related data (**Table 4**). The total numbers of infections reported were also remarkably higher in this study, suggesting possible geographic influence as well as methods/intent of data collection. Both developed TB despite a negative latent TB infection (LTBI) screen. Recently, a survey was conducted amongst physicians treating children with rheumatic disorders in India, that suggested a high incidence of TB, more so while the children were on biologics than after they were stopped.^34^ Thus, it seems here that what we see in Turkey is just the tip of the iceberg, and the problem might be much severe in areas of TB endemicity. In the current era of biosimilars, data from post marketing surveillance records in the developed world can be mined to gain insight into TB incidence rates.^35^

### Varied screening strategies before administering biologics

On a different note, low number of TB cases could also be due to varied TB and LTBI screening strategies before using bDMARDs. However, the recent survey from Indian rheumatologists suggests screening is universally practiced, though there is no consensus on the optimum method of screening.^34^ Thus, a closer look into the prevalent practices and cost-benefit ratios of the strategy used for screening might be insightful in the future. Recently, Hassanzadeh et al. established that blanket screening for TB using the TB Spot assay increased the risk of polypharmacy, adverse drug effects and increased cost manifold.^36^ Glasgow, an area of low TB prevalence and high BCG vaccination. Chest radiograph and clinical interview were used to identify risk factors for LTBI. The annual risk of TB was calculated using tables from BTS recommendations and then compared to the risk of drug-induced hepatitis. All patients were given a T-SPOT according to current local policy. Indeterminate T-SPOTs were recorded and repeated. Results . For 130 patients, a total of 160 tests were required resulting in a cost of £ 24,000. 99 (76% A recent systematic review confirmed the lack of consensus in screening strategies for TB in the immunosuppressed in guidelines across countries.^37^ Thus, region-specific data needs to be gathered before implementing screening strategies in rheumatology as the risk and cost efficacy ratios might differ significantly according to TB incidence rates.^37^

### Shorter follow-up duration in children

Moreover, studies can be marred by short follow-up period, as post-marketing surveillance offers best insight into rare adverse effects.^35^ Thus, registries are likely to provide a better overview. The PharmaChild registry which involved 32 countries across the globe reported 24 cases (17 on biological DMARDS) of tuberculosis in children with JIA.^38^ Similar compilations are particularly needed from parts the world with high background prevalence of tuberculosis. The short window of childhood might limit study periods as children move on to adulthood, as compared with studies in adults, which are likely to have longer follow-up periods.

### TB risk in children in comparison with adults

TB screening practices could vary in children, as can be the threshold to prescribe biologics. Varied Tuberculosis incidence in different regions call for region specific guidelines in screening keeping the risk benefit ratio in mind. Lack of clarity in current guidelines is likely to accentuate the problem.

### BCG vaccination

Difference in TB occurrence in children as compared with adults on anti-TNFs could also be a function of prevalent vaccination practices. Infant BCG vaccination has shown high efficacy of 70%–80% against childhood TB, especially meningeal and disseminated forms.^39^ Sara Suliman et al have shown that BCG re-vaccination in adults with LTBI induces long-lived BCG-reactive NK cell responses.^40^ This was in contrast to the limited cytokine change by Isoniazid preventive therapy, which was administered in 33 patients (39 in control group). Recently Katelaris et al. found that LTBI prevalence was lower amongst contacts of TB patients even 20 years after the initial vaccination, though vaccine efficacy declined as a function of time since vaccination.^41^ In light of waning vaccine efficacy in adulthood, BCG re-vaccination could possibly reduce TB incident rates while on bDMARDs.

## CONCLUSION

To conclude, there is dearth of data on incident TB rates in children with rheumatic disorders with exposure to bDMARDs from TB endemic countries. There is a felt need for regional registries to understand the prevalence, patterns, and prevalent screening practices to chalk out cost effective approaches with the intent to prevent long term debility.

## SUPPLEMENTARY TABLES

### Details of Selection of articles

#### Screening by title

The Scopus searches were imported into Zotero, and articles were first screened by title by one author, and those without relevance, systematic reviews, meta-analysis, narratives, and in languages other than English were removed (**Figure 1**).

#### Screening by abstract

The list of articles remaining after the initial screen was passed on to another author, to screen the individual abstracts for relevance, type of study and study population (children or adults). During the article screening, the initial rounds of elimination by screening titles and abstracts was done by one author each and subsequent rounds by two different authors.

#### Screening by reading full-text

The full text of articles obtained after two rounds of exclusions was then accessed. Those deemed irrelevant at this stage or where full-text was not available on the internet were then excluded. Similar screening strategy as delineated above was followed. The approach was mostly inclusive. Randomised placebo-controlled studies or any other controlled trials, retrospective case series, published data from registries, correspondence with data from more than 3 patients were included for data synthesis. Case reports, systematic reviews and metanalysis were excluded. Predesigned data extraction form (DEF), **Table 1** was used to record data from the articles obtained after the above three stages of screening. The DEF was devised by two rheumatologists individually who then discussed and merged the variables suggested by each.

Differences were resolved by consensus between two rheumatologists. DOI numbers, year and author names were recorded to avoid duplication of studies. TB was defined as in the individual studies.

Further, the number of studies from the various countries was tabulated and the number of participants, as well as TB cases, reported recorded for each. These were plotted on a world map (**Figures 1A,B**) to get a pictorial view of the geographic distribution of the data obtained. Multicentre studies were excluded from the above figure as attributions to individual countries were not possible. **Tables 1, 2 and 3** summarise data on tuberculosis in different paediatric rheumatic diseases, while **Table 4** summarises data obtained from various registries. Furthermore, the quality of evidence of each study was recorded and summarised as a **Table 2**, disease wise, to understand the weightage that can be accorded to each of these.

**Supplementary Table 1.** Data of tuberculosis in paediatric lupus and myositis on biologics.

**Retrospective**					
Drug	**RTX**	**RTX**	**RTX+CYC**	**RTX**	**RTX**
Country	Greece	Greece	Saudi-Arabia	Australia, CaNAda	CaNAda
Year	2011	2011	2013	2014	2015
Author	Maria TrachaNA^71^	Maria TrachaNA^71^	Ashwaq^72^	Dale^73^	M Olfat^74^
**Tuberculosis- No of patients**	**0**	**0**	**0**	**0**	**0**
Type of article/paper	Case series	Case series	Case series	Case series	Case series
N with complete data	4	4	16	18	24
Disease classification	SLE-LN	SLE-LN	SLE	NPSLE	Hematologic SLE
Duration of follow-up (Median, IQR, years)	1.33	1.33	3.2	2.5	3.6 (1.9–5.7)
Total no of infection events	0	0	2	NA	1
Major/serious Infections	0	0	2	NA	1
Number of events					
Opportunistic infections	0	0	NA	NA	NA
Minor Infections- Number of events	0	0	NA	NA	NA
Death			0	0	0
Biologic Doses received	375/m^2^, 4 doses	375/m^2^, 4 doses	375mg/m^2^, 2 doses	NA	375/m^2^, 4 doses
Duration of biologic treatment	One cycle	One cycle	One cycle for 12, 2 cycle for 2,4 cycles for 2. Each 6 months apart	NA	NA
Concomitant drugs	MMF (all)	MMF (all)	CYC, HCQ	NA	MMF (5), CYC (1)
Steroids	Yes (all)	Yes (all)	NA	Yes (all)	Yes, in 17
Portugal	USA	UK	USA	USA	Multicenter
2016	2015	2015	2015	2014	2013
Reis^75^4 with JSLE and 1 with extended oligoarticular JIA, received 10 cycles of RTX (23 infusions	Tambrelli^76^	Watson^77^	Hui yen^78^	Lehman^79^	Oddis^80^
**0**	**0**	**0**	**0**	**0**	**0**
Case series	Case series	Cohort	Cohort (adult and paediatric )	Cohort	randomized, placebo-phase controlled trial
5	104	63	39	12	48
SLE, JIA	50 SLE + 54 other AIRD	SLE	SLE	SLE-LN	JDM
2	2.2	NA	0.5	5	44 weeks
2	22	2	NA	2	NA^*^
2	20	2	7	2	NA^*^
1 (Cryptococcosis)	0	1 CMV	NA	NA	NA^*^
NA	2	NA	NA	NA	NA^*^
0	1 ILD	0	0	0	
750 mg, 2 doses	750 mg/m^2^ (maximum 1 g), administered 2 weeks apart	NA	750 mg/m^2^ administered twice 2 weeks apart	750mg/m^2^, 2 doses at 0,6,18 months	575 mg/m^2^ if BSA<1.5 m^2^ and 750 mg/m^2^ if BSA>1.5m^2^
NA	Median 2 (1–11) courses	NA	104 courses	18 months	NA
MMF (4)	MMF, CYC, HCQ	MMF, CYC, AZA (24)	MMF (49%), HCQ (92%), AZA (23%)	CYC	NA
Yes (all)	Yes, in all	Yes, in 93%	Yes, in 82%	Yes, in all	Yes, in all

RTX: Rituximab; CYC: Cyclophosphamide; USA: United States of America; UK: United Kingdom; SLE: Systemic lupus erythematosus; NPSLE: neuropsychiatric systemic lupus erythematosus; JIA: Juvenile idiopathic arthritis; LN: Lupus nephritis; AIIRD: Autoimmune inflammatory rheumatic diseases; IQR: Interquartile range; NA: Not available; CMV; Cytomegalovirus; ILD: Interstitial lung diseases; MMF: Mycophenolate mofetil; HCQ: hydroxychloroquine; AZA: Azathioprine; JDM: Juvenile dermatomyositis; BSA: Body surface area.

* *cannot differentiate between data from adult and juvenile DM*

**Supplementary Table 2.** Data of tuberculosis in paediatric vasculitis on biologics.

**Retrospective**						**Prospective**
**Drug**	**IFX 5** **ETN 1** **RTX 3**	**IFX 3** **ADA 2** **TCZ 3**	**IFX 9 ADA 1** **TCZ 2**	**TCZ 6**	**ADA-9**	**IFX 1**
Country	UK	UK	Canada	Turkey	Kazakhstan	Turkey
Year	2013	2015	2017	2018	2019	2017
Author	Despina Eleftheriou^81^	Despina Eleftheriou^82^	Florence A. Aeschlimann^83^	SezginSahin^84^	Dimitri Poddighe^85^	Nikos N. Markomichelakis^86^
**Tuberculosis-No of patients**	**0**	**0**	**0**	**0**	**0**	**0**
Type of article/paper	Single centre	Single tertiary referral centre	Single-centre cohort	Review of hospital records	Case-based review	Case series (adults and juvenile)
N with complete data	9	6	10	NA	9	1 (rest were adults)
Disease	Polyarteritis nodosa	Takayasu arteritis	Takayasu arteritis	Takayasu arteritis	Bechet’s Diseases	Behcet’s Disease
Duration of follow-up (Median, years)	3 (2.1–5)	NA	2.1 (IQR1.2–5.5)	NA	0.25–2	1
Total no of infection events	NA	NA	0	NA	NA	NA
Major/serious Infections-No of events	NA	NA	0	NA	NA	NA
Opportunistic infections	NA	NA	NA	NA	NA	NA
Minor Infections- No of events	NA	NA	NA	NA	NA	NA
Tuberculosis- No of patients	NA	NA	0	NA	0	0
Duration of biologic treatment (months)	NA	NA	Variable, 3–20	NA	3–24	3–24
Concomitant drugs	NA	NA	MTX (3), AZA (1)	NA	MMF (1), AZA (1)	AZA
Steroids	Yes, all	NA	Yes, in 3	NA	NA	Yes

IFX: Infliximab; ETN: Etanercept; RTX: Rituximab; ADA- Adalimumab; TCZ: Tocilizumab; UK: United Kingdom; USA: United States of America; IQR: Interquartile range; NA: Not available.

**Supplementary Table 3.** Data from paediatric biologic registries.

**Country**	Turkey	Thailand	Alabama, USA	Spain
**Year**	2017	2009	2017	2015
**Author**	Acar^87^	SuwanNAlai^88^	Stoll M^89^	Hernández^90^
**Tuberculosis- No of patients**	1 (JIA) on ADA	0	1 (IBD) on ADA	0
**Type of article/paper**	Retrospective analysis	Retrospective analysis of data from single centre	Retrospective analysis	Cohort observational study
**Total number**	N=73	N=5	N=1033	n=214
**Type of AIIRD**				
**JIA**	16 (21.9)	3	613	163 (73.6)
**SLE**	0	0	13	0
**Vasculitis (Including BD)**	3 (4.1)	0	5	0
**SSc/MCTD**	0	0	0	0
**Sarcoidosis**	3 (4.1)	0	17	0
**IIM**	0	1	3	0
**PSS**	0	0	7	0
**Uveitis**	39 (53.4)	0	31	8 (3.7)
**IBD**	8 (11)	0	265	46 (20.8)
**Autoinflammatory**	0	1	11	3 (1.5)
**Others**	4(5.5)	0	35	0
**Duration of follow-up (Median)**	18 (6–60) months	NA	1564 person-years	641 patients-year, Median- IQR 2.3 years (1.4–4.3).
**N total whose complete data is available**	73	5	1033	214
**Total no of infection events**	NA	NA	NA	NA
**Major/serious Infections- No of events**	NA	NA	NA	NA
**Opportunistic infections**	NA	NA	NA	NA
**Minor Infections- No of events**	NA	NA	NA	NA
**Drug**	**ADA-39** **ETN-22** **IFX-12**	**ETN-3** **IFX-2**	**IFX-527** **ADA-469** **ETN-324** **CER-9** **GOL-6**	**ETN-51.7%** **ADA (31.0 %)** **IFX-17.3%**
**Biologic Doses received**	NA	NA	NA	NA
**Duration of biologic treatment**	NA	NA	IFX- 840.6 ADA- 495.3 ETN-194.6 CER-2.0 GOL-1.5 Patient years	ETN 1.9 [1.8–3.7]; ADA 1.8 [1.2–2.6]; and IFX 2.1 [1.4–3.3] patient years
**Concomitant drugs**	MTX-37 (50.7) CYS-13 (17.8) AZA-9 (12.3)	NA	NA	NA
**Steroids**	45 (61.6)	NA	NA	NA
**Other**	NA	NA	NA	NA

**Supplementary Table 4.** Prevalence of tuberculosis in paediatric autoinflammatory diseases.

**Retrospective**				**Prospective**
**Country**	France	France	Italy	USA
**Year**	2012	2009	2010	2017
**Author**	Galeotti^91^	Neven^92^	Lepore^93^	Arostegui^94^
**Tuberculosis- No of patients**	**0**	**0**	0	0
**Type of article/paper**	E-mail survey among the members of the French Paediatric Society for Paediatric Rheumatology (SOFREMIP)-Registry based	Data from medical records of NOMID/CINCA syndrome patients from 2 centres	Registry based	Open label Phase II
**N with complete data**	6	8	**17**	
**Disease classification**	MKD n=6	NOMID/CINCA n=8	CINCA/MWS-n=17	HIDS n-6
**Duration of follow-up (Median, IQR, years)**	11–21 months	26–42 months	37.5 months (range, 12 to 54 months)	Max-24 months
**Total no of infection events**	2	0	NA	NA
**Major/serious Infections-Number of events**	1	0	NA	NA
**Opportunistic infections**	0	0	0	0
**Minor Infections-Number of events**	1	0	NA	NA
**Death**	0	0	0	0
**Drug**	**CAN-4** **ANK- 4**	**ANK-8**	**ANK-17**	**CAN-6**
**Biologic Doses received**	ANK-1 to 5 mg/kg/day CAN-2 to 7 mg/kg every 8 weeks	ANK- 3–10 mg/kf/day	ANK-starting dosage of 1 mg/kg/d (maximum, 100 mg)	300 mg (or4 mg/kg for patients weighing<40 kg)
**Duration of biologic treatment**	15 (4–72) months	26–42 months	NA	NA
**Concomitant drugs**	NA	NA	NA	NA
Steroids	1	NA	NA	NA

MKD: Mevalonate kinase deficiency; NOMID: Neonatal-onset multisystem inflammatory disease; CINCA: Chronic infantile neurologic, cutaneous, articular syndrome; MWS: Muckle Wells Syndrome; crFMF: Colchicine resistant familial Mediterranean fever; TRAPS: Tumor necrosis factor associated periodic fever; FCAS: familial cold autoinflammatory syndrome; HIDS: Hyperimmunoglobulinemia D with Periodic Fever Syndrome; RTX: Rituximab; CYC: Cyclophosphamide; USA: United States of America; UK: United Kingdom; SLE: Systemic lupus erythematosus; NPSLE: neuropsychiatric systemic lupus erythematosus; JIA: Juvenile idiopathic arthritis;

**Table:** 

UK	Multicenter Canada, USA, Germany, Ireland, Spain, Turkey, Switzerland, Russia, Japan	Multicenter	Germany	USA
2004	2018	2011	2011	2012
Hawkins^95^	Benedetti^96^	Kuemmerle-Deschner^97^	Kuemmerle-Deschner^98^	Sibley^99^
0	0	0	0	0
Prospective follow-up	Randomised controlled trial followed by an open label follow-up	open-label, phase III study conducted at 33 centres	Single centre observational study	Cohort-5 year follow-up
**1**	**53**	**46**	**5**	**20**
MWS-n=1	crFMF-n-14 MKD-n=28 TRAPS—n=14	FCAS-5 MWS-23 NOMID-18	MWS-5	NOMID-22
3 months	16 weeks	290 days (29–625 days)	11 months (range 5–14 months)	Max -5 years 148.1 patient-year
0	NA	NA	5	NA
0	8 cr-FMF-3/100 PY MKD-7/100 PY TRAPS-0/100 PY	NA	0	3
0	0	0	0	0
0	NA	NA	5	NA
0	0	0	0	0
**ANK-1**	CAN-56	**CAN-47**	**ANK-5**	**ANK-22**
ANK-100 mg once daily	CAN-150 mg, or 2 mg per kilogram of body weight for patients weighing ≤40 kg every week	150 mg or 2 mg/kg (≤40 kg) every 8 weeks for up to 2 years	1–2 mg/kg in patients weighing <40 kg and 100 mg for patients weighing >40 kg	started at 1 mg/kg by daily subcutaneous injection. Stepwise dose increases of 0.5–1 mg/kg per injection were made as frequently as every 2 weeks to achieve laboratory and organ inflammation remission
3 months	Exposure in PY crFMF- 45.6 MKD- 51.0 TRAPS-39.2	290 days (29–625 days)	At least 2 weeks	60 months
NA	Colchicine (100%)	NA	NA	NA
NA	NA	NA	NA	NA

LN: Lupus nephritis; AIIRD: Autoimmune inflammatory rheumatic diseases; IQR: Interquartile range; NA: Not available; CMV: Cytomegalovirus; ILD: Interstitial lung diseases; MMF: Mycophenolate mofetil; HCQ: hydroxychloroquine; AZA: Azathioprine.

## References

[1] Hiraki LT Feldman CH Marty FM Winkelmayer WC Guan H Costenbader KH. Serious Infection Rates Among Children With Systemic Lupus Erythematosus Enrolled in Medicaid. Arthritis Care Res 2017 Nov;69(11):1620–6. 10.1002/acr.23219 PMC5563276 28217919

[2] Muhammed H Gupta L Zanwar AA Misra DP Lawrence A Agarwal V Infections Are Leading Cause of In-Hospital Mortality in Indian Patients with Inflammatory Myopathy. J Clin Rheumatol 2019 Dec; 10.1097/RHU.0000000000001214 31804256

[3] Al-Mayouf SM Fallatah R Al-Twajery M Alayed T Alsonbul A. Outcome of children with systemic rheumatic diseases admitted to pediatric intensive care unit: An experience of a tertiary hospital. Int J Pediatr Adolesc Med 2019 Dec;6(4):142–5. 31890839 10.1016/j.ijpam.2019.07.003 PMC6926232

[4] TB India Report 2018: Ministry of Health and Family Welfare [Internet]. [cited 2020 Mar 27]. Available from: https://tbcindia.gov.in/showfile.php?lid=3314

[5] Gupta L Srivastava P Agarwal V Aggarwal A Able L Misra R High incidence of Tuberculosis in SLE patients [Internet]. Conference Abstract APLAR 2015 [cited 2020 Mar 27]. Available from: https://www.researchgate.net/publication/284448018_High_incidence_of_Tuberculosis_in_SLE_patients#fullTextFile-Content

[6] Gasparyan AY Ayvazyan L Blackmore H Kitas GD. Writing a narrative biomedical review: considerations for authors, peer reviewers, and editors. Rheumatol Int 2011 Nov 29;31(11):1409–17. 21800117 10.1007/s00296-011-1999-3

[7] Moorthy LN Peterson MG Hassett AL Lehman TJ. Burden of childhood-onset arthritis. Pediatr Rheumatol 2010 Dec 8;8(1):20–1. 10.1186/1546-0096-8-20 PMC2914068 20615240

[8] Hsin Y-C Zhuang L-Z Yeh K-W Chang C-W Horng J-T Huang J-L. Risk of Tuberculosis in Children with Juvenile Idiopathic Arthritis: A Nationwide Population-Based Study in Taiwan. Doherty TM, editor. PLOS ONE 2015 Jun 5;10(6):e0128768. 26047099 10.1371/journal.pone.0128768 PMC4457914

[9] Tuberculosis surveillance and monitoring in Europe 2016 [Internet]. [cited 2020 Mar 27]. Available from: http://www.euro.who.int/__data/assets/pdf_file/0019/310087/TB-surveillance-report-2016-Portugal.pdf?ua=1

[10] Sartori NS Picon P Papke A Neyeloff JL da Silva Chakr RM. A population-based study of tuberculosis incidence among rheumatic disease patients under anti-TNF treatment. Abu-Shakra M, editor. PLOS One. 2019 Dec 2;14(12):e0224963. 31790428 10.1371/journal.pone.0224963 PMC6886754

[11] Dixon WG Hyrich KL Watson KD Lunt M Galloway J Ustianowski A Drug-specific risk of tuberculosis in patients with rheumatoid arthritis treated with anti-TNF therapy: results from the British Society for Rheumatology Biologics Register (BSRBR). Ann Rheum Dis 2010 Mar;69(3):522–8. 19854715 10.1136/ard.2009.118935 PMC2927681

[12] Machuca I Vidal E de la Torre-Cisneros J Rivero-Román A. Tuberculosis en pacientes inmunodeprimidos. Enfermedades Infecc Microbiol Clínica 2018 Jun;36(6):366–74. 10.1016/j.eimc.2017.10.009 29223319

[13] Lee JY. Diagnosis and Treatment of Extrapulmonary Tuberculosis. Tuberc Respir Dis 2015;78(2):47. 10.4046/trd.2015.78.2.47 PMC4388900 25861336

[14] Lao M Chen D Wu X Chen H Qiu Q Yang X Active tuberculosis in patients with systemic lupus erythematosus from Southern China: a retrospective study. Clin Rheumatol 2019 Feb 23;38(2):535–43. 30244432 10.1007/s10067-018-4303-z

[15] Danza A Ruiz-Irastorza G. Infection risk in systemic lupus erythematosus patients: susceptibility factors and preventive strategies. Lupus 2013 Oct 4;22(12):1286–94. 24098001 10.1177/0961203313493032

[16] Pehlivan Y Kisacik B Bosnak VK Onat AM. Rituximab seems to be a safer alternative in patients with active rheumatoid arthritis with tuberculosis. Case Rep 2013 Jan 21;bcr2012006585–bcr2012006585. 10.1136/bcr-2012-006585 PMC3604500 23341582

[17] Marie I Hachulla E Chérin P Hellot M-F Herson S Levesque H Opportunistic infections in polymyositis and dermatomyositis. Arthritis Care Res 2005 Apr 15;53(2):155–65. 10.1002/art.21083 15818648

[18] Airio A Kauppi M Kautiainen H Hakala M Kinnula V. High association of mycobacterial infections with polymyositis in a non-endemic country for tuberculosis. Ann Rheum Dis 2007 Oct 1;66(10):1404–5. 17881668 10.1136/ard.2007.070177 PMC1994311

[19] Chen I-J Tsai W-P Wu Y-JJ Luo S-F Ho H-H Liou L-B Infections in polymyositis and dermatomyositis: analysis of 192 cases. Rheumatology 2010 Dec 1;49(12):2429–37. 20837496 10.1093/rheumatology/keq279

[20] Hernández-Cruz B Sifuentes-Osornio J Ponce-de-León Rosales S Ponce-de-León Garduño A Díaz-Jouanen E. Mycobacterium tuberculosis infection in patients with systemic rheumatic diseases. A case-series. Clin Exp Rheumatol 1999;17(3):289–96. 10410261

[21] Gattorno M Hofer M Federici S Vanoni F Bovis F Aksentijevich I Classification criteria for autoinflammatory recurrent fevers. Ann Rheum Dis 2019 Aug;78(8):1025–32. 31018962 10.1136/annrheumdis-2019-215048

[22] Levy R Gérard L Kuemmerle-Deschner J Lachmann HJ Koné-Paut I Cantarini L Phenotypic and genotypic characteristics of cryopyrin-associated periodic syndrome: a series of 136 patients from the Eurofever Registry. Ann Rheum Dis 2015 Nov;74(11):2043–9. 25038238 10.1136/annrheumdis-2013-204991

[23] Ozen S Demirkaya E Amaryan G Koné-Paut I Polat A Woo P Results from a multicentre international registry of familial Mediterranean fever: impact of environment on the expression of a monogenic disease in children. Ann Rheum Dis 2014 Apr;73(4):662–7. 23463692 10.1136/annrheumdis-2012-202708

[24] Lin PL Plessner HL Voitenok NN Flynn JL. Tumor Necrosis Factor and Tuberculosis. J Investig Dermatol Symp Proc 2007 May;12(1):22–5. 10.1038/sj.jidsymp.5650027 17502865

[25] Flynn JL Goldstein MM Chan J Triebold KJ Pfeffer K Lowenstein CJ Tumor necrosis factor-α is required in the protective immune response against mycobacterium tuberculosis in mice. Immunity 1995 Jun;2(6):561–72. 7540941 10.1016/1074-7613(95)90001-2

[26] Sia JK Rengarajan J. Immunology of Mycobacterium tuberculosis Infections. Microbiol Spectr 2019 Jul 5;7(4). 10.1128/microbiolspec.gpp3-0022-2018 PMC6636855 31298204

[27] Azfar SF Islam N. Suppression of Mycobacterium Tuberculosis Induced Reactive Oxygen Species andTumor Necrosis Factor-Alpha Activity in Human Monocytes of Systemic LupusErythematosus Patients by Reduced Glutathione. Oman Med J 2012 Jan 16;27(1):11–9. 22359719 10.5001/omj.2012.03 PMC3282130

[28] Yasui K. Immunity against Mycobacterium tuberculosis and the risk of biologic anti-TNF-α reagents. Pediatr Rheumatol 2014 Dec 2;12(1):45. 10.1186/1546-0096-12-45 PMC4196001 25317081

[29] Hsin Y-C Zhuang L-Z Yeh K-W Chang C-W Horng J-T Huang J-L. Risk of Tuberculosis in Children with Juvenile Idiopathic Arthritis: A Nationwide Population-Based Study in Taiwan. Doherty TM, editor. PLOS One 2015 Jun 5;10(6):e0128768. 26047099 10.1371/journal.pone.0128768 PMC4457914

[30] Zhu Z Kang Y Lin Z Huang Y Lv H Li Y. X-linked agammaglobulinemia combined with juvenile idiopathic arthritis and invasive Klebsiella pneumoniae polyarticular septic arthritis. Clin Rheumatol 2015 Feb 25;34(2):397–401. 24567239 10.1007/s10067-014-2537-y

[31] GARRED P RICHTER C ANDERSEN ÅB MADSEN HO MTONI I SVEJGAARD A Mannan-Binding Lectin in the Sub-Saharan HIV and Tuberculosis Epidemics. Scand J Immunol 1997 Aug 3;46(2):204–8. 9584002 10.1046/j.1365-3083.1997.d01-111.x

[32] Swart J Giancane G Horneff G Magnusson B Hofer M Alexeeva E Pharmacovigilance in juvenile idiopathic arthritis patients treated with biologic or synthetic drugs: combined data of more than 15,000 patients from Pharmachild and national registries. Arthritis Res Ther 2018 Dec 27;20(1):285. 30587248 10.1186/s13075-018-1780-z PMC6307151

[33] Swart J Giancane G Horneff G Magnusson B Hofer M Alexeeva E Pharmacovigilance in juvenile idiopathic arthritis patients treated with biologic or synthetic drugs: combined data of more than 15,000 patients from Pharmachild and national registries. Arthritis Res Ther 2018 Dec 27;20(1):285. 30587248 10.1186/s13075-018-1780-z PMC6307151

[34] Kavadichanda C Gupta L Balan S. Survey on Tuberculosis in children on biologics for rheumatic illnesses. Indian J Rheumatol 2020;15(2):130–3.

[35] Koike T Harigai M Ishiguro N Inokuma S Takei S Takeuchi T Safety and effectiveness of adalimumab in Japanese rheumatoid arthritis patients: postmarketing surveillance report of the first 3,000 patients. Mod Rheumatol 2012 Aug 13;22(4):498–508. 21993918 10.1007/s10165-011-0541-5

[36] Hassanzadeh R France J Bawa S. Targeted Screening for Latent TB Infection prior to Biologic Therapy to Improve Patient Safety and Reduce Costs: A Prospective Observational Study. ISRN Infect Dis 2014;201 4:1–6.

[37] Hasan T Au E Chen S Tong A Wong G. Screening and prevention for latent tuberculosis in immunosuppressed patients at risk for tuberculosis: a systematic review of clinical practice guidelines. BMJ Open 2018 Sep 12;8(9):e022445. 10.1136/bmjopen-2018-022445 PMC6144320 30209157

[38] Swart J Giancane G Horneff G Magnusson B Hofer M Alexeeva E Pharmacovigilance in juvenile idiopathic arthritis patients treated with biologic or synthetic drugs: combined data of more than 15,000 patients from Pharmachild and national registries. Arthritis Res Ther 2018 Dec 27;20(1):285. 30587248 10.1186/s13075-018-1780-z PMC6307151

[39] Trunz BB Fine P Dye C. Effect of BCG vaccination on childhood tuberculous meningitis and miliary tuberculosis worldwide: a meta-analysis and assessment of cost-effectiveness. The Lancet 2006 Apr;367(9517):1173–80. 10.1016/S0140-6736(06)68507-3 16616560

[40] Suliman S Geldenhuys H Johnson JL Hughes JE Smit E Murphy M Bacillus Calmette–Guérin (BCG) Revaccination of Adults with Latent Mycobacterium tuberculosis Infection Induces Long-Lived BCG-Reactive NK Cell Responses. J Immunol 2016 Aug 15;197(4):1100–10. 27412415 10.4049/jimmunol.1501996 PMC4976036

[41] Katelaris AL Jackson C Southern J Gupta RK Drobniewski F Lalvani A Effectiveness of BCG Vaccination Against Mycobacterium tuberculosis Infection in Adults: A Cross-sectional Analysis of a UK-Based Cohort. J Infect Dis 2020 Jan 1;221(1):146–55. 31504674 10.1093/infdis/jiz430

[42] Bracaglia C Buonuomo PS Tozzi AE Pardeo M Nicolai R Campana A Safety and Efficacy of Etanercept in a Cohort of Patients with Juvenile Idiopathic Arthritis Under 4 Years of Age. J Rheumatol 2012 Jun 15;39(6):1287–90. 22589254 10.3899/jrheum.111555

[43] Favalli EG Pontikaki I Becciolini A Biggioggero M Ughi N Romano M Real-life 10-year retention rate of first-line anti-TNF drugs for inflammatory arthritides in adult- and juvenile-onset populations: similarities and differences. Clin Rheumatol 2017 Aug 8;36(8):1747–55. 28597133 10.1007/s10067-017-3712-8

[44] Ayaz NA Demirkaya E Bilginer Y Özçelik U Çobanoğlu N Kiper N Preventing tuberculosis in children receiving anti-tnf treatment. Clin Rheumatol 2010 Apr 19;29(4):389–92. 20084445 10.1007/s10067-009-1334-5

[45] Hugle B Burgos-Vargas R Inman RD O’Shea F Laxer RM Stimec J Long-term outcome of anti-tumor necrosis factor alpha blockade in the treatment of juvenile spondyloarthritis. Clin Exp Rheumatol 32(3):424–31. 24387974

[46] Brunelli JB Bonfiglioli KR Silva CA Kozu KT Goldenstein-Schainberg C Bonfa E Latent tuberculosis infection screening in juvenile idiopathic arthritis patients preceding anti-TNF therapy in a tuberculosis high-risk country. Rev Bras Reumatol Engl Ed 2017 Sep;57(5):392–6. 29037310 10.1016/j.rbre.2016.11.004

[47] Kilic O Kasapcopur O Camcioglu Y Cokugras H Arisoy N Akcakaya N. Is it safe to use anti-TNF-α agents for tuberculosis in children suffering with chronic rheumatic disease? Rheumatol Int 2012 Sep 26;32(9):2675–9. 21789614 10.1007/s00296-011-2030-8

[48] Zuber Z Rutkowska-Sak L Postępski J Dobrzyniecka B Opoka-Winiarska V Kobusińska K Etanercept treatment in juvenile idiopathic arthritis: The Polish registry. Med Sci Monit 2011;17(12):SR35–42. 22129916 10.12659/MSM.882109 PMC3628139

[49] Verazza S Davì S Consolaro A Bovis F Insalaco A Magni-Manzoni S Disease status, reasons for discontinuation and adverse events in 1038 Italian children with juvenile idiopathic arthritis treated with etanercept. Pediatr Rheumatol 2016 Dec 20;14(1):68. 10.1186/s12969-016-0126-0 PMC5170898 27993144

[50] Saini Isha Lesa Dawman NG *SK K. Biologicals in Juvenile Idiopathic Arthritis. Indian Pediatr 2016;(53):260–1. 27029697

[51] Atikan BY Cavusoglu C Dortkardesler M Sozeri B. Assessment of tuberculosis infection during treatment with biologic agents in a BCG-vaccinated pediatric population. Clin Rheumatol 2016 Feb 18;35(2):427–31. 25515621 10.1007/s10067-014-2842-5

[52] Aygun D Sahin S Adrovic A Barut K Cokugras H Camcıoglu Y The frequency of infections in patients with juvenile idiopathic arthritis on biologic agents: 1-year prospective study. Clin Rheumatol 2019 Apr 17;38(4):1025–30. 30448935 10.1007/s10067-018-4367-9

[53] Mourão AF Santos MJ Melo Gomes JA Martins FM Mendonça SC Oliveira Ramos F Effectiveness and long-term retention of anti-tumour necrosis factor treatment in juvenile and adult patients with juvenile idiopathic arthritis: data from Reuma.pt. Rheumatology 2016 Apr;55(4):697–703. 26672905 10.1093/rheumatology/kev398

[54] Horneff G Foeldvari I Minden K Trauzeddel R Kümmerle-Deschner JB Tenbrock K Efficacy and Safety of Etanercept in Patients With the Enthesitis-Related Arthritis Category of Juvenile Idiopathic Arthritis: Results From a Phase III Randomized, Double-Blind Study. Arthritis Rheumatol 2015 May;67(8):2240–9. 25891010 10.1002/art.39145

[55] Giannini EH Ilowite NT Lovell DJ Wallace CA Rabinovich CE Reiff A Long-term safety and effectiveness of etanercept in children with selected categories of juvenile idiopathic arthritis. Arthritis Rheum 2009 Sep;60(9):2794–804. 19714630 10.1002/art.24777

[56] Prince FHM Twilt M Cate R ten van Rossum MAJ Armbrust W Hoppenreijs EPAH Long-term follow-up on effectiveness and safety of etanercept in juvenile idiopathic arthritis: the Dutch national register. Ann Rheum Dis 2009 May;68(5):635–41. 18413443 10.1136/ard.2007.087411

[57] Ruperto N Lovell DJ Quartier P Paz E Rubio-Pérez N Silva CA Long-term safety and efficacy of abatacept in children with juvenile idiopathic arthritis. Arthritis Rheum 2010 Feb 26;62(6):1792–802. 20191582 10.1002/art.27431

[58] Constantin T Foeldvari I Vojinovic J Horneff G Burgos-Vargas R Nikishina I Two-year Efficacy and Safety of Etanercept in Pediatric Patients with Extended Oligoarthritis, Enthesitis-related Arthritis, or Psoriatic Arthritis. J Rheumatol 2016 Apr;43(4):816–24. 26932344 10.3899/jrheum.150430

[59] Imagawa T Yokota S Mori M Miyamae T Takei S Imanaka H Safety and efficacy of tocilizumab, an anti-IL-6-receptor monoclonal antibody, in patients with polyarticular-course juvenile idiopathic arthritis. Mod Rheumatol 2012 Feb 12;22(1):109–15. 21667343 10.1007/s10165-011-0481-0

[60] Brunelli JB Schmidt AR Sallum AME Goldenstein-Schainberg C Bonfá E Silva CA High rate of serious infection in juvenile idiopathic arthritis patients under biologic therapy in a real-life setting. Mod Rheumatol 2018 Mar 4;28(2):264–70. 28949278 10.1080/14397595.2017.1349059

[61] Tarkiainen M Tynjälä P Vähäsalo P Lahdenne P. Occurrence of adverse events in patients with JIA receiving biologic agents: long-term follow-up in a real-life setting. Rheumatology 2015 Jul;54(7):1170–6. 25504896 10.1093/rheumatology/keu457

[62] Kingsbury DJ Bader-Meunier B Patel G Arora V Kalabic J Kupper H. Safety, effectiveness, and pharmacokinetics of adalimumab in children with polyarticular juvenile idiopathic arthritis aged 2 to 4 years. Clin Rheumatol 2014 Oct 2;33(10):1433–41. 24487484 10.1007/s10067-014-2498-1 PMC4161937

[63] Imagawa T Takei S Umebayashi H Yamaguchi K Itoh Y Kawai T Efficacy, pharmacokinetics, and safety of adalimumab in pediatric patients with juvenile idiopathic arthritis in Japan. Clin Rheumatol 2012 Dec 2;31(12):1713–21. 23053683 10.1007/s10067-012-2082-5 PMC3505492

[64] Lovell DJ Reiff A Ilowite NT Wallace CA Chon Y Lin S-L Safety and efficacy of up to eight years of continuous etanercept therapy in patients with juvenile rheumatoid arthritis. Arthritis Rheum 2008 May;58(5):1496–504. 18438876 10.1002/art.23427

[65] Lovell DJ Ruperto N Goodman S Reiff A Jung L Jarosova K Adalimumab with or without Methotrexate in Juvenile Rheumatoid Arthritis. N Engl J Med 2008 Aug 21;359(8):810–20. 18716298 10.1056/NEJMoa0706290

[66] Ruperto N Lovell DJ Cuttica R Wilkinson N Woo P Espada G A randomized, placebo-controlled trial of infliximab plus methotrexate for the treatment of polyarticular-course juvenile rheumatoid arthritis. Arthritis Rheum 2007 Sep;56(9):3096–106. 17763439 10.1002/art.22838

[67] Klein A Becker I Minden K Foeldvari I Haas J Horneff G. Adalimumab versus adalimumab and methotrexate for the treatment of juvenile idiopathic arthritis: long-term data from the German BIKER registry. Scand J Rheumatol 2019 Mar 4;48(2):95–104. 30411654 10.1080/03009742.2018.1488182

[68] Southwood TR Foster HE Davidson JE Hyrich KL Cotter CB Wedderburn LR Duration of etanercept treatment and reasons for discontinuation in a cohort of juvenile idiopathic arthritis patients. Rheumatology 2011 Jan 1;50(1):189–95. 21047801 10.1093/rheumatology/keq308

[69] Schmeling H Minden K Foeldvari I Ganser G Hospach T Horneff G. Efficacy and Safety of Adalimumab as the First and Second Biologic Agent in Juvenile Idiopathic Arthritis: The German Biologics JIA Registry. Arthritis Rheumatol 2014 Sep;66(9):2580–9. 24942886 10.1002/art.38741

[70] Horneff G Schulz AC Klotsche J Hospach A Minden K Foeldvari I Experience with etanercept, tocilizumab and interleukin-1 inhibitors in systemic onset juvenile idiopathic arthritis patients from the BIKER registry. Arthritis Res Ther 2017 Dec 22;19(1):256. 29166924 10.1186/s13075-017-1462-2 PMC5700562

[71] Sevcic K Orban I Brodszky V Bazso A Balogh Z Poor G Experiences with tumour necrosis factor- inhibitors in patients with juvenile idiopathic arthritis: Hungarian data from the National Institute of Rheumatology and Physiotherapy Registry. Rheumatology 2011 Jul 1;50(7):1337–40. 21372001 10.1093/rheumatology/ker103

[72] Otten MH Prince FHM Twilt M ten Cate R Armbrust W Hoppenreijs EPAH Tumor Necrosis Factor-blocking Agents for Children with Enthesitis-related Arthritis — Data from the Dutch Arthritis and Biologicals in Children Register, 1999–2010. J Rheumatol 2011 Oct;38(10):2258–63. 21844151 10.3899/jrheum.110145

[73] Kearsley-Fleet L Sampath S McCann LJ Baildam E Beresford MW Davies R Use and effectiveness of rituximab in children and young people with juvenile idiopathic arthritis in a cohort study in the United Kingdom. Rheumatology 2019 Feb 1;58(2):331–5. 30358861 10.1093/rheumatology/key306 PMC6343463

[74] Trachana M Koutsonikoli A Farmaki E Printza N Tzimouli V Papachristou F. Safety and efficacy of Rituximab in refractory pediatric systemic lupus erythematosus nephritis: a single-center experience of Northern Greece. Rheumatol Int 2013 Mar 19;33(3):809–13. 22101555 10.1007/s00296-011-2239-6

[75] AlE’ed A AlSonbul A Al-Mayouf SM. Safety and efficacy of combined cyclophosphamide and rituximab treatment in recalcitrant childhood lupus. Rheumatol Int 2014 Apr 12;34(4):529–33. 24218286 10.1007/s00296-013-2896-8

[76] Dale RC Brilot F Duffy LV. Twilt M Waldman AT Narula S. Utility and safety of rituximab in pediatric autoimmune and inflammatory CNS disease. Neurology 2014 Jul 8;83(2):142–50. 24920861 10.1212/WNL.0000000000000570 PMC4117174

[77] Olfat M Silverman ED Levy DM. Rituximab therapy has a rapid and durable response for refractory cytopenia in childhood-onset systemic lupus erythematosus. Lupus 2015 Aug 24;24(9):966–72. 25804672 10.1177/0961203315578764

[78] Reis J Aguiar F Brito I. Anti CD20 (Rituximab) therapy in refractory pediatric rheumatic diseases. Acta Reumatol Port 41(1):45–55. 27115107

[79] Tambralli A Beukelman T Cron RQ Stoll ML. Safety and Efficacy of Rituximab in Childhood-onset Systemic Lupus Erythematosus and Other Rheumatic Diseases. J Rheumatol 2015 Mar;42(3):541–6. 25593242 10.3899/jrheum.140863

[80] Watson L Beresford MW Maynes C Pilkington C Marks SD Glackin Y The indications, efficacy and adverse events of rituximab in a large cohort of patients with juvenile-onset SLE. Lupus 2015 Jan 12;24(1):10–7. 25117653 10.1177/0961203314547793

[81] Hui-Yuen JS Reddy A Taylor J Li X Eichenfield AH Bermudez LM Safety and Efficacy of Belimumab to Treat Systemic Lupus Erythematosus in Academic Clinical Practices. J Rheumatol 2015 Dec;42(12):2288–95. 26523030 10.3899/jrheum.150470 PMC5031077

[82] Lehman TJ Singh C Ramanathan A Alperin R Adams A Barinstein L Prolonged improvement of childhood onset systemic lupus erythematosus following systematic administration of rituximab and cyclophosphamide. Pediatr Rheumatol 2014 Dec 14;12(1):3. 10.1186/1546-0096-12-3 PMC3896732 24423147

[83] Oddis C V. Reed AM Aggarwal R Rider LG Ascherman DP Levesque MC. Rituximab in the treatment of refractory adult and juvenile dermatomyositis and adult polymyositis: A randomized, placebo-phase trial. Arthritis Rheum 2013 Feb;65(2):314–24. 23124935 10.1002/art.37754 PMC3558563

[84] Eleftheriou D Dillon MJ Tullus K Marks SD Pilkington CA Roebuck DJ Systemic Polyarteritis Nodosa in the Young: A Single-Center Experience Over Thirty-Two Years. Arthritis Rheum 2013 Sep;65(9):2476–85. 23754739 10.1002/art.38024

[85] Eleftheriou D Varnier G Dolezalova P McMahon A-M Al-Obaidi M Brogan PA. Takayasu arteritis in childhood: retrospective experience from a tertiary referral centre in the United Kingdom. Arthritis Res Ther 2015 Dec 25;17(1):36. 25879697 10.1186/s13075-015-0545-1 PMC4392477

[86] Aeschlimann FA Eng SWM Sheikh S Laxer RM Hebert D Noone D Childhood Takayasu arteritis: disease course and response to therapy. Arthritis Res Ther 2017 Dec 22;19(1):255. 29166923 10.1186/s13075-017-1452-4 PMC5700506

[87] Sahin S Hopurcuoglu D Bektas S Belhan E Adrovic A Barut K Childhood-onset Takayasu arteritis: A 15-year experience from a tertiary referral center. Int J Rheum Dis 2019 Jan;22(1):132–9. 30397997 10.1111/1756-185X.13425

[88] Poddighe D Mukusheva Z Dauyey K Assylbekova M. Adalimumab in the treatment of pediatric Behçet’s disease: case-based review. Rheumatol Int 2019 Jun 11;39(6):1107–12. 30976833 10.1007/s00296-019-04300-0

[89] Markomichelakis NN Aissopou EK Maselos S Tugal-Tutkun I Sfikakis PP. Biologic Treatment Options for Retinal Neovascularization in Behçet’s Disease. Ocul Immunol Inflamm 2019 Jan 2;27(1):51–7. 28700280 10.1080/09273948.2017.1332228

[90] Acar M Sütçü M Aktürk H Hançerli-Torun S Erol OB Salman N Tuberculosis screening in pediatric patients receiving tnf-alpha inhibitor therapy. Turk J Pediatr 2017;59(5):503. 29745110 10.24953/turkjped.2017.05.001

[91] Suwannamalai P Authavekiat P Udomsubpayakul U Janavitayanujit S. The infectious profiles of anti-tumor necrosis factor agents in a Thai population: a retrospective study a the university-based hospital. Int J Rheum Dis 2009 Jul;12(2):118–24. 20374328 10.1111/j.1756-185X.2009.01393.x

[92] Stoll ML Grubbs JA Beukelman T Mannion ML Jester TW Cron RQ Risk of tuberculosis among Alabama children and adolescents treated with tumor necrosis factor inhibitors: a retrospective study. Pediatr Rheumatol 2017 Dec 9;15(1):79. 10.1186/s12969-017-0207-8 PMC5679346 29121953

[93] Calzada-Hernández J Anton-López J Bou-Torrent R Iglesias-Jiménez E Ricart-Campos S Martín de Carpi J Tuberculosis in pediatric patients treated with anti-TNFα drugs: a cohort study. Pediatr Rheumatol 2015 Dec 3;13(1):54. 10.1186/s12969-015-0054-4 PMC4669612 26635208

[94] Galeotti C Meinzer U Quartier P Rossi-Semerano L Bader-Meunier B Pillet P Efficacy of interleukin-1-targeting drugs in mevalonate kinase deficiency. Rheumatology 2012 Oct 1;51(10):1855–9. 22740624 10.1093/rheumatology/kes097

[95] Neven B Marvillet I Terrada C Ferster A Boddaert N Couloignier V Long-term efficacy of the interleukin-1 receptor antagonist anakinra in ten patients with neonatal-onset multisystem inflammatory disease/chronic infantile neurologic, cutaneous, articular syndrome. Arthritis Rheum 2010 Jan;62(1):258–67. 20039428 10.1002/art.25057

[96] Lepore L Paloni G Caorsi R Alessio M Rigante D Ruperto N Follow-Up and Quality of Life of Patients with Cryopyrin-Associated Periodic Syndromes Treated with Anakinra. J Pediatr. 2010 Aug;157(2):310–15.e1. 20472245 10.1016/j.jpeds.2010.02.040

[97] Arostegui JI Anton J Calvo I Robles A Iglesias E López-Montesinos B Open-Label, Phase II Study to Assess the Efficacy and Safety of Canakinumab Treatment in Active Hyperimmunoglobulinemia D With Periodic Fever Syndrome. Arthritis Rheumatol 2017 Aug;69(8):1679–88. 28482144 10.1002/art.40146

[98] Hawkins PN Lachmann HJ Aganna E McDermott MF. Spectrum of clinical features in Muckle-Wells syndrome and response to anakinra. Arthritis Rheum 2004 Feb;50(2):607–12. 14872505 10.1002/art.20033

[99] De Benedetti F Gattorno M Anton J Ben-Chetrit E Frenkel J Hoffman HM Canakinumab for the Treatment of Autoinflammatory Recurrent Fever Syndromes. N Engl J Med 2018 May 17;378(20):1908–19. 29768139 10.1056/NEJMoa1706314

[100] Kuemmerle-Deschner JB Hachulla E Cartwright R Hawkins PN Tran TA Bader-Meunier B Two-year results from an open-label, multicentre, phase III study evaluating the safety and efficacy of canakinumab in patients with cryopyrin-associated periodic syndrome across different severity phenotypes. Ann Rheum Dis 2011 Dec 1;70(12):2095–102. 21859692 10.1136/ard.2011.152728

[101] Kuemmerle-Deschner JB Tyrrell PN Koetter I Wittkowski H Bialkowski A Tzaribachev N Efficacy and safety of anakinra therapy in pediatric and adult patients with the autoinflammatory Muckle-Wells syndrome. Arthritis Rheum 2011 Mar;63(3):840–9. 21360513 10.1002/art.30149

[102] Sibley CH Plass N Snow J Wiggs EA Brewer CC King KA Sustained response and prevention of damage progression in patients with neonatal-onset multisystem inflammatory disease treated with anakinra: A cohort study to determine three- and five-year outcomes. Arthritis Rheum 2012 Jul;64(7):2375–86. 22294344 10.1002/art.34409 PMC3474541

